# Arterial Mechanical Motion Estimation Based on a Semi-Rigid Body Deformation Approach

**DOI:** 10.3390/s140609429

**Published:** 2014-05-27

**Authors:** Pablo Guzman, Ghassan Hamarneh, Rafael Ros, Eduardo Ros

**Affiliations:** 1 Department of Computer Archiecture and Technology, ETSI Informática y de Telecomunicación, CITIC-UGR, University of Granada, 18071 Granada, Spain; E-Mail: eros@ugr.es; 2 School of Computing Science, Simon Fraser University, Burnaby, BC V5A 1S6, Canada; E-Mail: hamarneh@sfu.ca; 3 Hospital Universitario San Cecilio, Servicio de Angiología y Cirugía Vascular, 18008 Granada, Spain; E-Mail: rafael.ros.sspa@juntadeandalucia.es

**Keywords:** computer vision, ultrasound, wall motion, arterial stiffness, elastography, carotid, motion analysis

## Abstract

Arterial motion estimation in ultrasound (US) sequences is a hard task due to noise and discontinuities in the signal derived from US artifacts. Characterizing the mechanical properties of the artery is a promising novel imaging technique to diagnose various cardiovascular pathologies and a new way of obtaining relevant clinical information, such as determining the absence of dicrotic peak, estimating the Augmentation Index (AIx), the arterial pressure or the arterial stiffness. One of the advantages of using US imaging is the non-invasive nature of the technique unlike Intra Vascular Ultra Sound (IVUS) or angiography invasive techniques, plus the relative low cost of the US units. In this paper, we propose a semi rigid deformable method based on Soft Bodies dynamics realized by a hybrid motion approach based on cross-correlation and optical flow methods to quantify the elasticity of the artery. We evaluate and compare different techniques (for instance optical flow methods) on which our approach is based. The goal of this comparative study is to identify the best model to be used and the impact of the accuracy of these different stages in the proposed method. To this end, an exhaustive assessment has been conducted in order to decide which model is the most appropriate for registering the variation of the arterial diameter over time. Our experiments involved a total of 1620 evaluations within nine simulated sequences of 84 frames each and the estimation of four error metrics. We conclude that our proposed approach obtains approximately 2.5 times higher accuracy than conventional state-of-the-art techniques.

## 1. Introduction

Estimating the variation of motion in the artery for vascular characterization [1] is a new technique that helps doctors to detect specific diseases. Other non-invasive techniques such as Ankle Brachial Pressure Index (ABPI) [2] or Augmentation Index (AIx) have been used to estimate parameters (blood pressure) that are associated with peripheral vascular diseases. For example, Mortensen *et al.* [3] demonstrated the relation of AIx and the Marfan syndrome, the role that involves AIx in the hypertension field [4] and the increase of the arterial stiffness in human subjects with Type 1 diabetes mellitus [5]. The way to estimate the pressure parameters becomes very limited due to the fact that such measures cannot be estimated in other parts of the body besides the carotid artery where we have an easy access with US. Arterial pressure and arterial wall motion are related since estimating the pressure requires measuring the variation of the diameter of the artery, as it is indicated in Equation (2). The importance of the wall motion artery's characterization has been also discussed by several authors who have demonstrated that radial [1,6–8] and longitudinal [9–11] motion are promising indicators to be associated with certain diseases or pathologies.

Existing commercial solutions such as Tissue Doppler Imaging (TDI) focus on the velocity measurement of the myocardial motion using Doppler principles. This technique has been extended to other applications in echocardiography [1,6], to determine the mechanical properties of vessels by means of TDI. The main problems with using TDI are that the motion vector measurement can only be done in parallel to the direction of the ultrasound beam, TDI measures absolute tissue velocity, and it is not able to distinguish all passive motion [12].

Different solutions have been proposed to characterize the wall artery motion directly from ultrasound images in order to complement the information about motion patterns extracted from B-mode US. Image intensity correlation techniques have been widely used in ultrasound due to their robustness under noisy environments. Golemati *et al.* [13] compared the displacement error produced in block matching [14] and optical flow [15,16] methods over a simulated dataset. The matching feature is also an important factor, where Soleimani [17] demonstrates that by including the gradient in the local search, the method results improve. The inclusion of a Kalman filter [18] to update the reference block and the displacement vector [19–21] has been also evaluated. This method becomes useful when the registered data is corrupted by significant amounts of noise, but in cases where the information is not corrupted at all, the filter does not improve the accuracy of the system rather produces over-smoothing. Other authors [22,23] go one step further and not only measure the displacement of the wall, but also include the Pulse Wave Velocity (PWV) to estimate the pressure by mean Moens-Korteweg Equation (1), that relates the PWV to the elasticity of the arterial wall:

(1) $$\text{PWV}=\sqrt{\frac{A_{0}}{\rho }\frac{\partial P}{\partial A}}$$

where *A*_o_ is the arterial diameter in the diastole, *ρ* is the density of blood, ∂*A* is the variation of the diameter over time (determined using cross-correlation in [22,23]) with respect to the artery in resting position, and ∂*P* is the difference in the pressure with respect to the end-diastolic pressure *P*(0). The pressure produced in the artery can be estimated using *PWV*, as shown in Equation (2):

(2) $$\partial P=\frac{\rho \cdot {\text{PWV}}^{2}}{A_{0}}\partial A$$

Compared to previous works, our method supports sub-pixel accuracy and incorporate collective motion information to define the wall artery motion. The major contribution of this paper is the evaluation of different methods and how they can be integrated to better address our problem of estimating the change in diameter ∂*A*. In this work, in order to enhance existing motion tracking methods, a combination of similarity transformation, non-rigid deformations, statistical filtering, and hybrid motion estimation techniques are proposed. In this way, it will be possible to estimate useful parameters instead of using more expensive and invasive methods that put the patient's well-being at risk.

The paper is organized as follows: Section 2 introduces a detailed explanation of the evaluated models, the process to generate the ground truth estimation and the combination of the methods that will be evaluated and compared in Section 3. In Section 4, the obtained results of the evaluated methods will be discussed and finally, Section 5 summarizes some conclusions and outlines for proposed future work.

## 2. Material and Methods

In this section, the methodological ‘building blocks’ used in this paper will be first briefly described. Then, the performance of the methods in different analysis pipelines will be evaluated.

### 2.1. Evaluated Methods

#### 2.1.1. Block Matching

The block matching (BM) technique has been a very popular method in the ultrasound field because it provides a robust estimation of the motion by means of comparing the similarity between blocks of different images. One of the uses of motion estimation via BM technique is the one proposed by Basarab [24], where the elastography map is estimated to show hidden objects such as cysts or cancer tumors in ultrasound imaging. This work uses a multiscale scheme to avoid errors in the motion estimation and to obtain a low sub-pixel resolution. It is important to remark that normalized cross correlation (NCC) block matching method is one of the most popular techniques utilized in ultrasound tracking [9–11,22,25]. In our evaluation, it was decided to make use of Lewis method [26] due to the fact that the obtained performance is much superior to the original one (approximately 15 times faster). Lewis method consists of a modification of the NCC technique where the similarity is given by Equation (3):

(3) $$\frac{\sum_{\left(i,j\right)\in W}\left(I_{1}\left(i,j\right)-\bar{I_{1}}\right)\cdot \left(I_{2}\left(x+i,y+j\right)-\bar{I_{2}}\right)}{\sqrt[{2}]{\sum_{\left(i,j\right)\in W}{\left(I_{1}\left(i,j\right)-\bar{I_{1}}\right)}^{2}\cdot \sum_{\left(i,j\right)\in W}{\left(I_{2}\left(x+i,y+j\right)-\bar{I_{2}}\right)}^{2}}}$$

where *I*_1_ is the reference block with size *W*, *I*_2_ is the image where the correlation is carried out, *I*_1̅_ and *I*_2̅_ are their respective means. The numerator in Equation (3) can be sees as a convolution (as in Equation (4)) between two images, *f* (*i, j*) = *I*_1_(*i, j*) − *I*_1̅_ and *t* (*i, j*) = *I*_2_(*i, j*) − *I*_2̅_, and can be efficiently solved by means of a Fourier convolution:

(4) $$\sum_{\left(i,j\right)\in W}f\left(i,j\right)\cdot t\left(x+i,y+j\right)$$

On the other hand, the denominator of Equation (3) must be solved efficiently. Lewis [26] proposed the use of the integral of the image technique to compute it efficiently and reduce the computation cost.

#### 2.1.2. Optical Flow

The temporal variation in an ordered sequence of images allows the estimation of the optical flow 2D vector, usually denoted as *v⃗* = (*u*, *v*), and is computed based on the constant-brightness hypothesis, which assumes that the pixel brightness remains constant over time. This leads to the formulation of the famous optical flow constraint Equation (5):

(5) $$uf_{x}+vf_{y}+f_{t}=0$$

where *u* and *v* are the optical flow components and the spatio-temporal derivates are represented by *f_x_*, *f_y_* and *f_t_* respectively. It is important to remark that in the two considered optical flow methods (described in Sections 2.1.3 and 2.1.4), the texture domain of the image was used, as proposed by Wedel *et al.* [27], so as to avoid problems with the brightness assumption.

#### 2.1.3. Optical Flow: Lucas and Kanade

On the basis of the optical flow constraint equation, Lucas and Kanade [15] proposed the minimization of the error Equation (6) using the sum of the least squares:

(6) $$E\left(u,v\right)={\sum_{i\in B}\left(f_{x}\left(i\right)u+f_{y}\left(i\right)v+f_{t}\left(i\right)\right)}^{2}$$

Equation (6) is minimized by taking the partial derivatives with respect to the optical flow vector v⃗. The resulting *v⃗* minimizes the differential error between the previous image and the current image and is given is presented in Equation (7):

(7) $$\vec{v}={\left[\begin{array}{cc} \underset{i\in B}{\text{∑}}f_{x}^{2}\left(i\right) & \underset{i\in B}{\text{∑}}f_{x}\left(i\right)f_{y}\left(i\right)\\ \underset{i\in B}{\text{∑}}f_{x}\left(i\right)f_{y}\left(i\right) & \underset{i\in B}{\text{∑}}f_{y}^{2}\left(i\right) \end{array}\right]}^{-1}\left[\begin{array}{c} -\underset{i\in B}{\text{∑}}f_{x}\left(i\right)f_{t}\left(i\right)\\ -\underset{i\in B}{\text{∑}}f_{y}\left(i\right)f_{t}\left(i\right) \end{array}\right]$$

where *v⃗* is the optical flow vector, the spatio-temporal derivates are represented by *f_x_*, *f_y_* and *f_t_* respectively, and the subscript *i* is the *i-th* element of the integration block *B*.

#### 2.1.4. Optical Flow: Anisotropic TV-L1

The anisotropic optical flow Equation (8), proposed by Werlberger *et al.* [28], is an extension of the popular method TV-L1 optical flow [29], which is based on a regularized propagation technique similar to the one proposed by Horn and Schunck [16]. Werlberger proposes an anisotropic diffusor that does not propagate values through the edges, with better preserves image structure. In this case, the original formulation was changed in order to reduce the computation cost in the last term of Equation (8), where possible artifacts (e.g., occlusions) can be rectified over the time incorporating feedback from previous optical flow:

(8) $$\min_{\vec{u},\vec{v}}\sup_{\left|{\vec{p}}_{d}\right|\leq 1}\left\{\int_{\Omega }\sum_{d=1}^{2}\left[\left(D^{½}\nabla u_{d}\right)\cdot {\vec{p}}_{d}-\varepsilon \frac{{\left|{\vec{p}}_{d}\right|}^{2}}{2}+\frac{1}{2\theta }{\left(u_{d}-v_{d}\right)}^{2}\right]+\delta \left|\rho \left(\vec{v}\left(\vec{x}\right)\right)\right|+\frac{\lambda }{2}\int_{\Omega }{\left(u\prime_{d}-u_{d}\right)}^{2}d\vec{x}\right\}$$

where $D^{½}=\exp\left(-\alpha {\left|\nabla I\right|}^{\beta }\right)\vec{\text{n}}{\vec{\text{n}}}^{T}+{\vec{\text{n}}}^{⊥}{\vec{\text{n}}}^{⊥T}$, $\vec{\text{n}}=\frac{\nabla I}{\left|\nabla I\right|}$as the normal vector, and n⃗^⊥^ the tangent vector of a given point. *u _d_* is the optical flow vector and *u′_d_* is the previous warped optical flow [30,31].

#### 2.1.5. Kalman Filter

In noisy systems, the Kalman Filter [18] has been proposed due to its robustness and efficiency. This method is based on a statistical approach to determine the current estimation of a linear system from a collection of previous observations over time as described in Equation (9):

(9) $${\hat{x}}_{k}=A{\hat{x}}_{k-1}+Bu_{k-1}+w_{k-1}$$

where *x̂* is the predicted estimation in the current time k, *x̂_k_*_−1_ is the previous observation, *A* and *B* describe the transition and control matrix respectably, *u_k_*_−1_ is the control signal, and *w_k_*_−1_, the process noise of the system.

#### 2.1.6. Similarity Transformation

Incorporating shape prior knowledge has become common practice in segmentation methods in the last decades. Cootes *et al.* [32] proposes a statistical method able to deform a contour by means of weighting relevant eigenvectors (*P*) by shape parameters (*b*) with the objective of adapting the contour to a desired object in the image, as shown in Equation (10):

(10) $$x=\bar{x}+P\cdot b$$

The objective of this method is estimating the shape parameters as well as the pose parameters (translation in x and y-axis, scale, rotation) that locate the desired object to be segmented in Equation (11):

(11) $$v=\left\{t_{x},t_{y},s,\theta ,b\right\}$$

These parameters are found by means of matching each landmark with the previous trained normalized gradient profiles and solving linear equations [33]. In our work, we will not adopt the statistically-based deformations, thus the local deformation terms can be discarded. In other words, we neglect the shape variability encoded in *b* and focus on estimating the remaining pose parameters only. Therefore, Equation (10) is no longer needed in our approach and we only utilize the weighted similarity transformation proposed in [33]. Such a similarity transformation is obtained by means of the weighted sum minimization in Equation (12):

(12) $$E={\left(x1-M\left(s,\theta \right)\left[x2\right]-t\right)}^{T}W\left(x1-M\left(s,\theta \right)\left[x2\right]-t\right)$$

where:

(13) $$\begin{array}{c} M\left(s,\theta \right)\left[\begin{array}{c} x\\ y \end{array}\right]=\left(\begin{array}{c} \left(s\cdot \cos\theta \right)\cdot x-\left(s\cdot \sin\theta \right)\cdot y\\ \left(s\cdot \sin\theta \right)\cdot x+\left(s\cdot \cos\theta \right)\cdot y \end{array}\right),\\ t={\left(t_{x},t_{y},\ldots ,t_{x},t_{y}\right)}^{T} \end{array}$$

*x*1 is the origin point and *x*2 the translated point, *s* the scale, *θ* the rotation, *t* the translation vector, and W is a diagonal matrix of weights for each point.

#### 2.1.7. Soft Body Dynamics

Soft body models have been widely used in computer science to carry out realistic physical simulations of motion and deformable objects. Rather than a statistically-based deformation model in Equation (10), this paper will focus on the popular mass-spring model, which is based on a mesh of nodes (masses) and connected by means of elastic links (springs). The basis of this method relies on Hooke's law, to simulate the spring force, and the second Newton's law to simulate the dynamics by time integration. In this work, a simplification of the idea proposed by Hamarneh *et al.* [33] will be adopted. The authors describe a system in Equation (14) that involves forces generated by the springs' system, in Equation (15), in a controlled environment (Equation (16)) allowing for speedup/slowdown of the velocity of the mesh's nodes:

(14) $$f_{i}=f_{i}^{\text{Hooke}}+f_{i}^{\text{Viscous}}$$

(15) $$f_{i}^{\text{Hooke}}=-k_{s}\left(\left\|x_{i}-x_{j}\right\|-r_{ij}\right)\frac{x_{i}-x_{j}}{\left\|x_{i}-x_{j}\right\|}-\left(k_{d}{\left(v_{i}-v_{j}\right)}^{T}\frac{x_{i}-x_{j}}{\left\|x_{i}-x_{j}\right\|}\right)\frac{x_{i}-x_{j}}{\left\|x_{i}-x_{j}\right\|}$$

(16) $$f_{i}^{\text{Viscous}}=-k_{v}v_{i}$$

where *f_i_* is the final estimated force, *k_s_* the Hooke's spring constant, *x_i_* the Cartesian coordinate of the *i-th* node, *r_ij_* the rest length associated to a link between two nodes, *k_d_* the damping constant, *k_v_* the viscosity coefficient, and *v_i_* as the velocity of the *i-th* node. Once the nodes' force is obtained, it is possible to estimate the acceleration, velocity, and position of each node by means of the iterative scheme in Equation (17):

(17) $$\begin{array}{c} a_{i}=\frac{f_{i}}{m_{i}}\\ v_{i}=v_{i}^{\text{old}}+a_{i}\Delta t\\ x_{i}=x_{i}^{\text{old}}+v_{i}\Delta t \end{array}$$

where *a_i_* is the acceleration, *f_i_* the force described in Equation (14), *v_i_* the velocity, *x_i_* the position, and Δ*t* the time interval.

### 2.2. Ground Truth Estimation via Ultrasound Simulation

**Figure 1.**
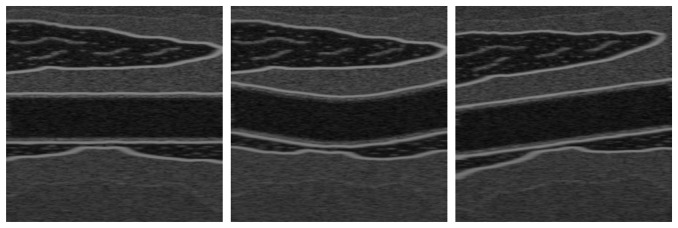
Ultrasound images used in the data set test, generated by Field II U.S. simulator.

To evaluate the proposed methods, the Field II Ultrasound MATLAB library [34] was used to generate nine sequences simulating the wall displacement of the common carotid artery. Each sequence involves a complete cycle of the cardiac system with a frequency of 25 Hz per cycle. These simulations were generated with 1024 physical elements, a transducer center frequency of 5 MHz, 100 MHz of sampling frequency, and 64 active elements. The sequences are based on three different topologies as shown in Figure 1, where diverse amplitudes of motion are used, as explained later in this section. Although these synthetic images are clearer than real US artery images, having the ground-truth motion allows quantitative evaluation metrics to compare different methods. We include also the simulation parameters used to produce these synthetic sequences (Table 1) to facilitate the reproduction of our results.

Following the steps of Stoitsis *et al.* [35], that describe a mathematical mechanical deformation model of the arterial wall as a separable method in space and time, this paper will simulate the radial and longitudinal displacement of the artery by means of Equations (18) and (19), which is a simplification of the original method.

(18) $$rt\left(t\right)=\prod \left(t_{0},t_{1}\right)\cdot \sin^{2}\frac{\pi \cdot t}{c\cdot T}+\prod \left(t_{1},t_{2}\right)\cdot \left(a+b\cdot t\right)$$

(19) $$\prod \left(t_{i},t_{j}\right)=f\cdot \left(1+\tanh\left(d\cdot \left(t-t_{i}\right)\right)\right)\cdot \left(1+\tanh\left(d\cdot \left(t_{j}-t\right)\right)\right)$$

where *a* and *f* determinate the amplitude of the waveform, b defines the slope in the second part of the curve, *c* and *T* are coefficients that control the initial part of the curve, *d* determines the wall artery speed, *t*_1_ and *t*_2_ correspond to the duration of the first and second pulse of the waveform and *t* is the time variable. In the generated data set, the chosen parameters are shown in Table 1, where different values of *f* let us control the amplitude of the artery displacement. For each generated artery topology, three values of *f* will be applied to produce different radial and longitudinal displacements.

### 2.3. Proposed Methods

Following the introduction of the methodological building blocks in the previous section, the proposed combination of methods to be evaluated in Section 3 will be explained in detail. To estimate the motion of the wall of the artery, two optical flow methods and the block matching approach proposed by Lewis [26] will be evaluated.

**Figure 2.**
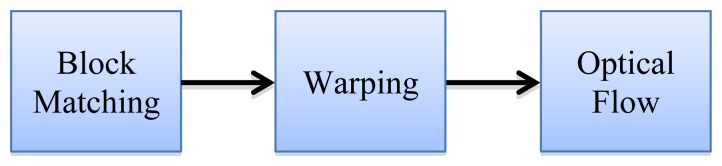
Block matching with sub-pixel accuracy by means of the optical flow scheme.

One of the main problems in classical block matching techniques is the sub-pixel accuracy. To handle this problem in an efficient way, other authors [36,37] proposed a combination of optical flow and block matching to increase the motion vector precision. This approach relies on estimating firstly the motion vector by means of the block matching technique, and then applying a warping [30,31] to the block and computing the optical flow to estimate the sub-pixel information (as shown in Figure 2).

**Figure 3.**
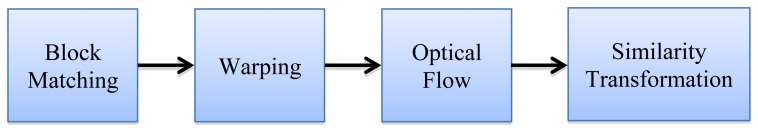
Similarity transformation given the motion vectors obtained with the hybrid BM-optical flow method.

Most of the wall artery tracking papers take into account the tracking individually [13,20,21] but do not consider all the tracking points as a set of data that define a semi-rigid object in motion. In this paper we use all tracked points when estimating the similarity transformation parameters (rotation, scale, and translation), as explained in Section 2.1. This method obtains all the motion vectors for all point and estimates the transformation parameters by means of Equation (13). One of the advantages of this method is the robustness against noise, allowing the computation of the parameters that define the transformation even with some wrong (or outlier) motion vectors. Figure 3 describes the steps that define this proposed scheme.

**Figure 4.**
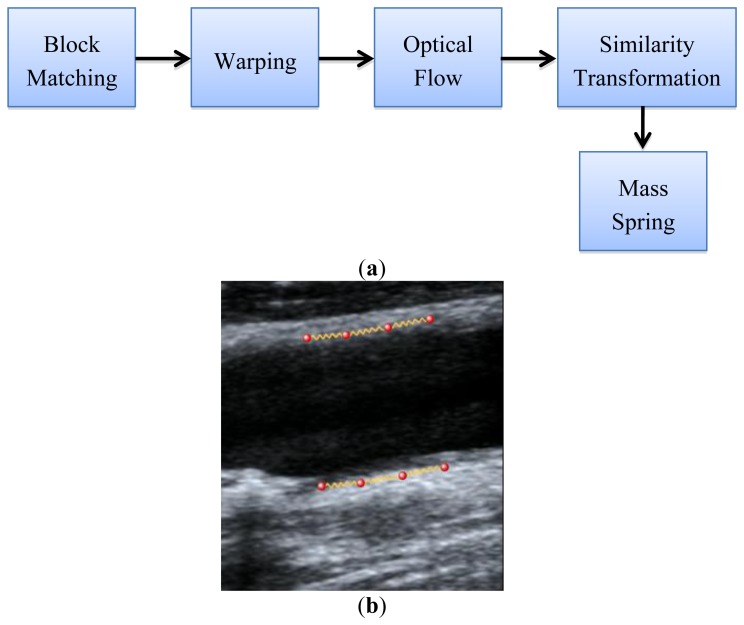
(**a**) Pipeline of the proposed method with physics simulation (mass-spring) and (**b**) Illustration of the spring connections in an ultrasound image.

When the similarity transformation is applied, it produces an error in the position transformation. This artifact happened because the method considers the systolic-diastolic-systolic transition as a scale-translation transformation over time. To reduce this error, rather than using the statistical shape deformation model of Equation (10), a physically-based simulation method (Soft Body, Section 2.1) could be included to the upper and lower set of points (individually) that track the artery, as indicated in Figure 4a. The pipeline of this approach is illustrated in Figure 4b.

**Figure 5.**
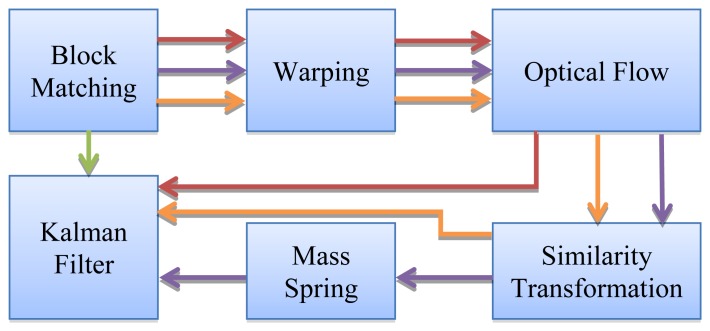
Incorporation of Kalman Filter in the previously proposed schemes. Each scheme is encoded with a different color.

Finally, the KF (Kalman Filter, Section 2.1) will be incorporated into this evaluation. This filter was previously used by Gastounioti *et al.* [20,21] to increase the accuracy of tracking the wall artery tracking. In this particular case, the KF will be incorporated into different proposed schemes as described by Figure 5 with the objective of being evaluated a posteriori in Section 3 as well as the other proposed schemes. It is important to note that in our evaluations a simple updating scheme was added to update the reference block (the initial tracked block) in each frame of the sequence, as shown in Equation (20):

(20) $$B_{\text{new}\_\text{ref}}=\alpha \cdot B_{\text{old}\_\text{ref}}+\left(1-\alpha \right)\cdot B_{\text{estimated}}$$

where *B_new_*___*_ref_* is the new estimated reference block, *B_old_*___*_ref_* the old referenced block, *B_estimated_* is the estimated displaced block, and *α* is the parameter that controls the amount of information that remains from the old reference block; we empirically set *α* to 0.98.

## 3. Results

As described in Section 2.2, we generated a set of nine sequences that simulate the wall artery motion with different amplitudes and topologies. The displacement of the wall of the artery, in our evaluation set, varies from 2.50 up to 9 pixels. To evaluate the proposed methods in Section 2.3, a similar metric as the one used by Golemati *et al.* [13] will be used. In our evaluation, each sequence contained 84 frames and involved three cardiac cycles. To obtain an impartial evaluation, six different positions (a total of 54 evaluations per method) will be evaluated in each sequence and the Cartesian coordinate error Equations (21)–(23) and the diameter error over the time Equation (24) will be measured by mean of root-mean-squared error (RMSE):

(21) $${\bar{\varepsilon }}_{\text{long}}=\sqrt{\frac{1}{N\cdot M}\sum_{j=0}^{M-1}\sum_{i=0}^{N-1}{\left(x\left(i,j\right)-x\prime \left(i,j\right)\right)}^{2}}$$

(22) $${\bar{\varepsilon }}_{\text{rad}}=\sqrt{\frac{1}{N\cdot M}\sum_{j=0}^{M-1}\sum_{i=0}^{N-1}{\left(y\left(i,j\right)-y\prime \left(i,j\right)\right)}^{2}}$$

(23) $${\bar{\varepsilon }}_{p}=\sqrt{\frac{1}{N\cdot M}\sum_{j=0}^{M-1}\sum_{i=0}^{N-1}\left({\left\|p\left(i,j\right)-p\prime \left(i,j\right)\right\|}^{2}\right)}$$

(24) $${\bar{\varepsilon }}_{d}=\sqrt{\frac{1}{\frac{N}{2}\cdot M}\sum_{j=0}^{M-1}\sum_{i=0}^{\frac{N}{2}-1}{\left(d\left(i,j\right)-d\prime \left(i,j\right)\right)}^{2}}$$

where *x*(*i*, *j*), *y*(*i*, *j*) and *p*(*i*, *j*) are the *i-th* x, y and (x,y) coordinates in the *j-th* frame of the sequence with its respective ground truth *x′*(*i*, *j*), *y′*(*i*, *j*), *p′*(*i*, *j*) and *d*(*i*, *j*) is the estimated diameter of the artery and its ground truth *d′*(*i*, *j*). N is the number of points, in our case, six points, and M is the number of frames per sequence. Our evaluation uses five motion models: Lucas and Kanade, Anisotropic Huber L1, BM (Lewis method), BM + Lucas & Kanade, and BM + Anisotropic Huber TV-L1. For each motion model, the methods described in Section 2.3 will be used. These methods include the similarity transformation (ST), the Kalman filter (KF), and the mass-spring (MS) physics based model. The motion methods will also be evaluated individually as shown in Table 2.

**Figure 6.**
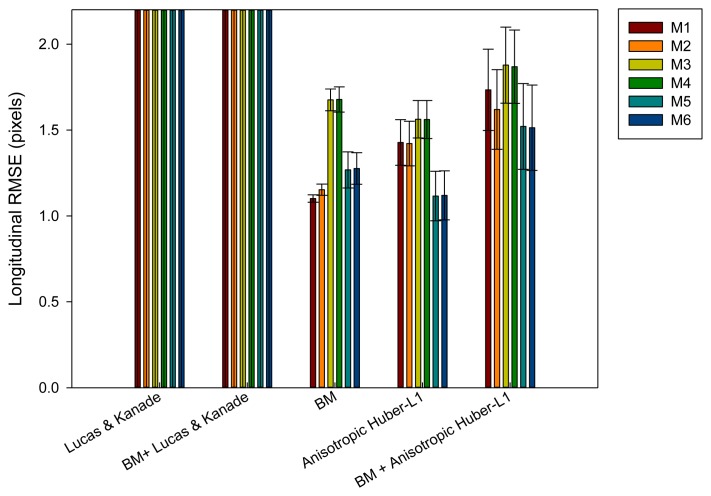
Longitudinal error results after being evaluated with different methods.

At first, the estimated longitudinal error will be evaluated by means of Equation (21), in our evaluation set (Section 2.2). As can be appreciated in Figure 6, the methods that make use of the Lucas and Kanade algorithm generate the highest number of errors. This is due to the fact that this method does not handle properly the aperture problem compared to the other motion evaluated methods. M1 with block matching and M5 method with anisotropic TV-L1 motion estimation produce the best results in the longitudinal motion estimation. In some plots the errors values are above the maximum values of the plot. We have reduced the plot range to better discriminate among the other approaches.

**Figure 7.**
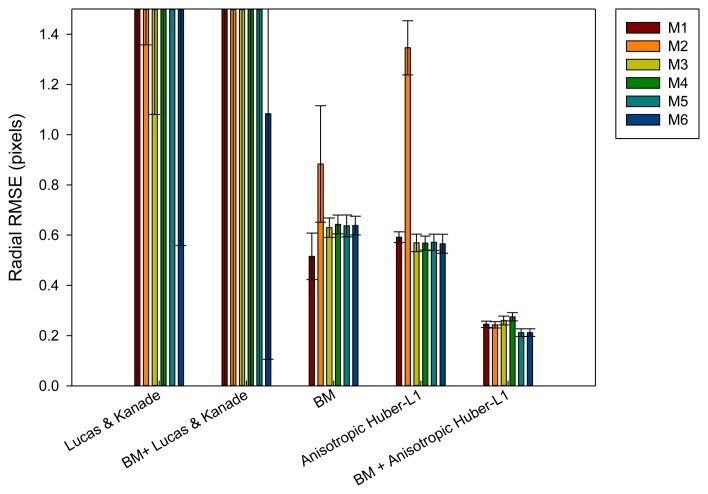
Radial error results after being evaluated with different methods.

In the radial motion evaluation (Figure 7) computed by means of Equation (22), the methods with Lucas and Kanade obtain again worse results. But unlike the longitudinal evaluation, the mix of block matching and anisotropic TV-L1 obtain the best results and a lower deviation with respect to the best results of the block matching approaches, achieving almost 50% less error. It is important to remark that in both evaluations (longitudinal and radial), the inclusion of the mass-spring method (M5–M6) helps, in general, to reduce the position error.

**Figure 8.**
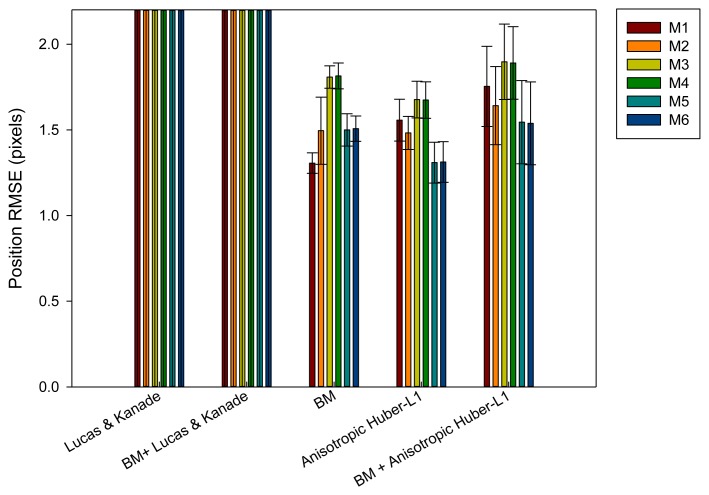
Position error results after being evaluated with different methods.

A global position evaluation (Figure 8) is carried out by Equation (23), where the radial and longitudinal displacement is taken into account. In general, the block matching (M1 version) and anisotropic TV-L1 methods achieve the best results with the difference that BM generates less standard deviation in the error among different evaluated sequences.

**Figure 9.**
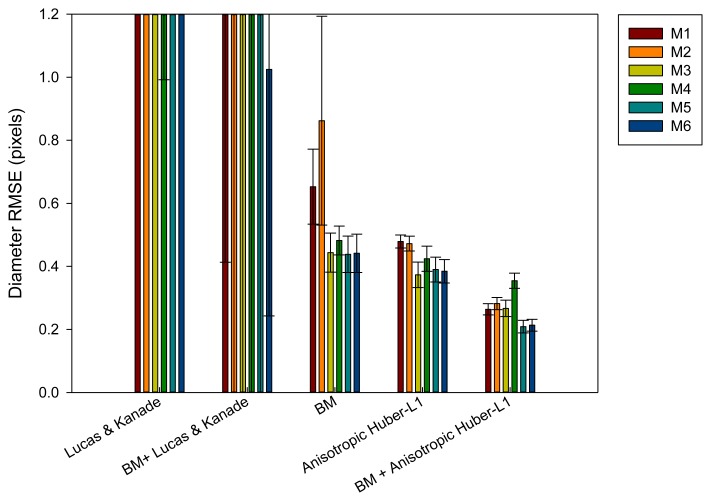
Diameter error results after being evaluated with different methods.

It is important to obtain a method capable of achieving good position precision. In our case, it is not critical to estimate the evolution of the diameter of the artery over time with a model that generates a small deviation on the elastomer's position (±1 pixel). To evaluate the most significant parameter, the diameter of the elastomer over time, estimations obtained from Equation (24) will be evaluated in our assessment set (Figure 9). The results reveal that the best method is the combination of block matching and anisotropic TV-L1 (M5). If we compare it with the previous results that evaluate the position error, the best solution (BM-M1) generates 3.1 times higher error than the new best solution obtained. The combination of optical flow and block matching obtain almost two times higher precision than the methods working individually.

After evaluating the different proposed approaches, it can be observed that the inclusion of the Kalman filter (M2, M4 and M6) provides worse results with respect to the other approaches. The methods that include the similarity transformation reduce the error up to 14% in some proposed approaches. After incorporating the soft body model, an increase of 13% in the precision is obtained in the last method (BM and anisotropic TV-L1). Finally, it can be concluded that a combination of optical flow and block matching and the M5 scheme becomes the most precise technique to estimate the desired parameter.

**Figure 10.**
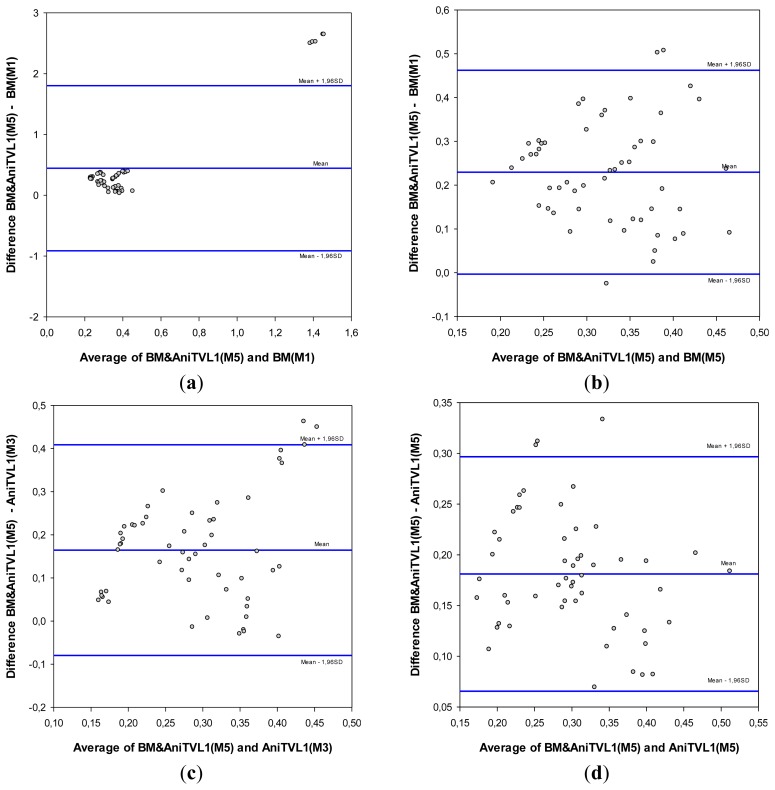
Bland-Altman figure, where the best obtained results block matching & anisotropic TV-L1 (M5) are compared with (**a**) block matching (M1), (**b**) block matching (M5), (**c**) anisotropic TV-L1 (M3), and (**d**) anisotropic TV-L1 (M5).

To show a more detailed evaluation between the best method and other results, a Bland-Altman plot is produced. Figure 10 shows the difference between the best method with other evaluated methods and their averages. The middle line indicates the average difference of both methods, whereas the upper and lower lines represent 95% limits of agreement with 15.80% (Figure 10a), 49.78% (Figure 10b), 33.66% (Figure 10c), and 78.39% (Figure 10d) of window (defined by 1.96 times the standard deviation with respect to the mean difference) displacement with respect to the origin coordinate. It can be concluded from Figure 10 that there is an overall good agreement of the amplitudes between the BM and anisotropic TV-L1 (M5) method and the reference ones. To facilitate the evaluation, further tabulated results are listed in the appendix. In the next section, the obtained results will be discussed and the method will be evaluated in real cases with different human subjects *in vivo*, with the objective of validating this technique.

## 4. Discussion

**Figure 11.**
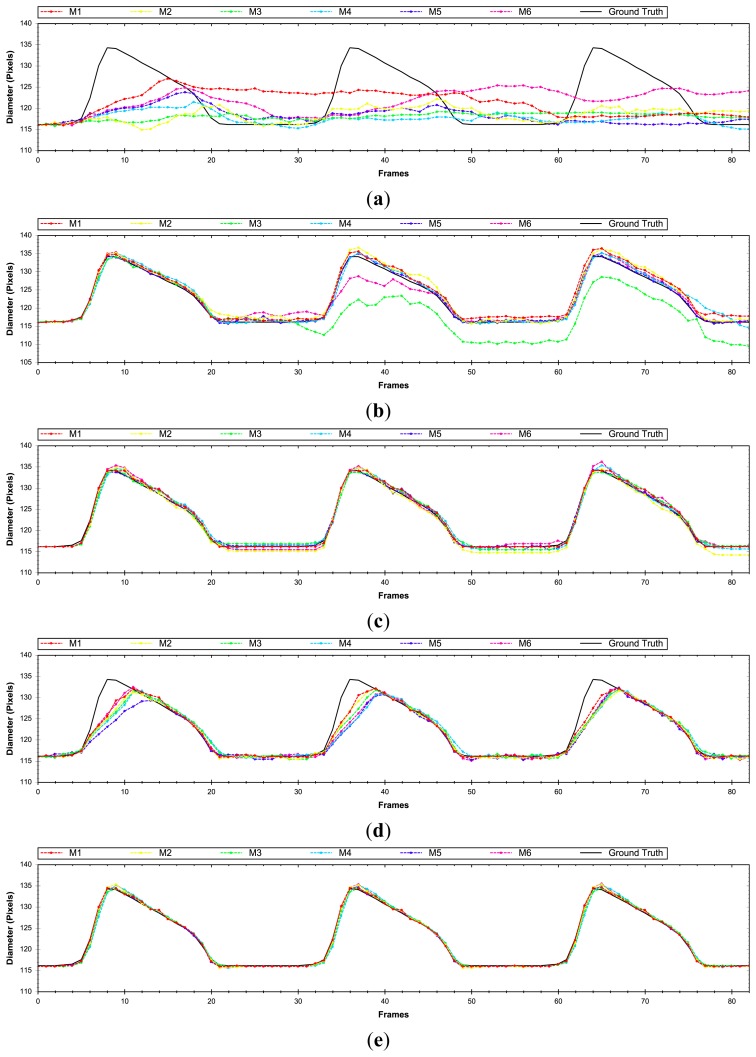
Diameter evolution over time evaluated in a sequence with long displacements using (**a**) Lucas-Kanade, (**b**) block matching & Lucas-Kanade, (**c**) block matching, (**d**) anisotropic TV-L1, and (**e**) block matching & anisotropic TV-L1.

**Figure 12.**
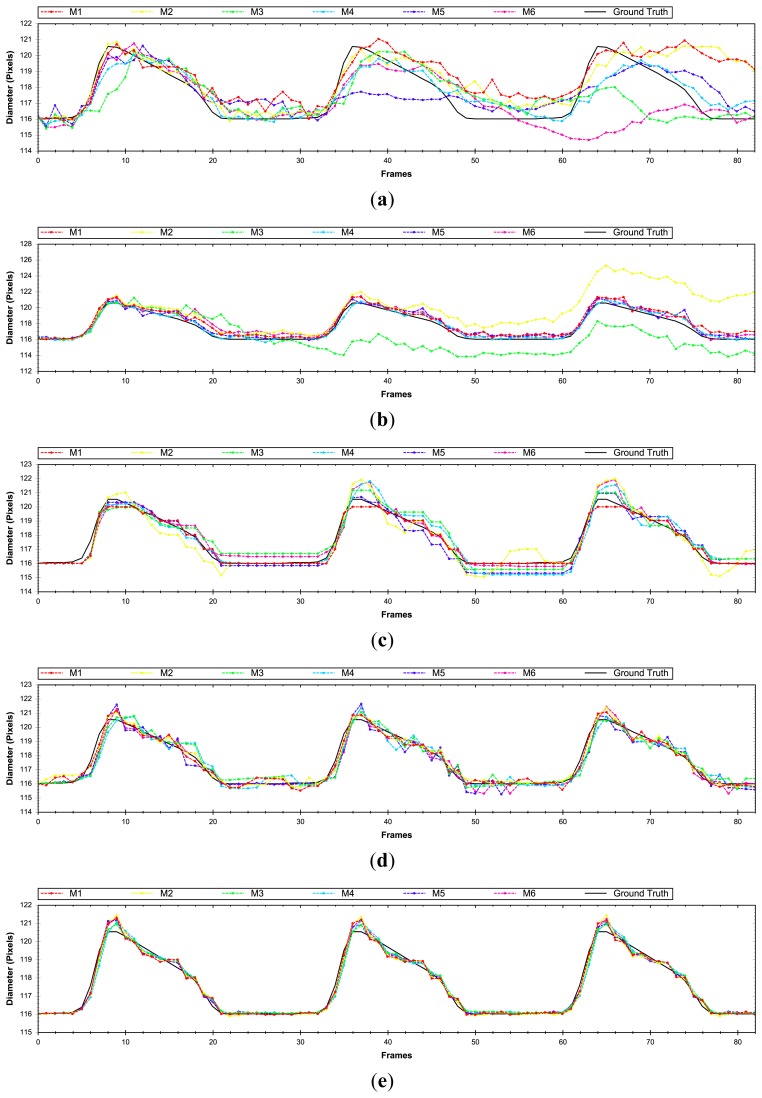
Diameter evolution over time evaluated in a sequence with small displacements evaluated with (**a**) Lucas-Kanade, (**b**) Block Matching & Lucas-Kanade, (**c**) Block Matching, (**d**) Anisotropic TV-L1, and (**e**) Block Matching & Anisotropic TV-L1.

In this section, the proposed approaches and the impact of each method in different evaluations will be discussed. Figures 11 and 12 show the proposed evaluations in two different sequences, in order to illustrate the response of the methods under different motion variations.

Figure 11 corresponds to a simulation that produces a maximum displacement of 18 pixels (the variation in displacement between the upper and lower arterial wall), while Figure 12 generates a maximum wall displacement of 5 pixels. As was discussed in the previous section, the Lucas and Kanade method is not the most appropriate algorithm to register the motion in US images as illustrated in Figures 11a,b and 12a,b. When displacement vectors are long, the optical flow based techniques are not the most convenient ones, because these methods have a maximum limit to determine the motion vector (thus improving the working range would require multiscale schemes such as [38]) as illustrated in Figure 11d.

Moreover, Block Matching techniques do not have this limitation, but produce rough results and allow no sub-pixel precision (Figures 11 and 12c). A good solution is the combination of correlation and optical flow techniques to avoid this displacement limitation and obtain sub-pixel precision (Figures 11 and 12e), acquiring twice more precision than with only block matching method (approx. 0.25 pixel error), but with the inconvenience of increased computation time. The inclusion of Kalman filters does not significantly increase or decrease the results, but it is interesting to include it in hypothetical cases when the system has a severe disturbance and noise. The main problem with the use of this filter is that, depending on the settings of the parameters, the signal may be over smoothed and shifted in relation with the desired one.

**Figure 13.**
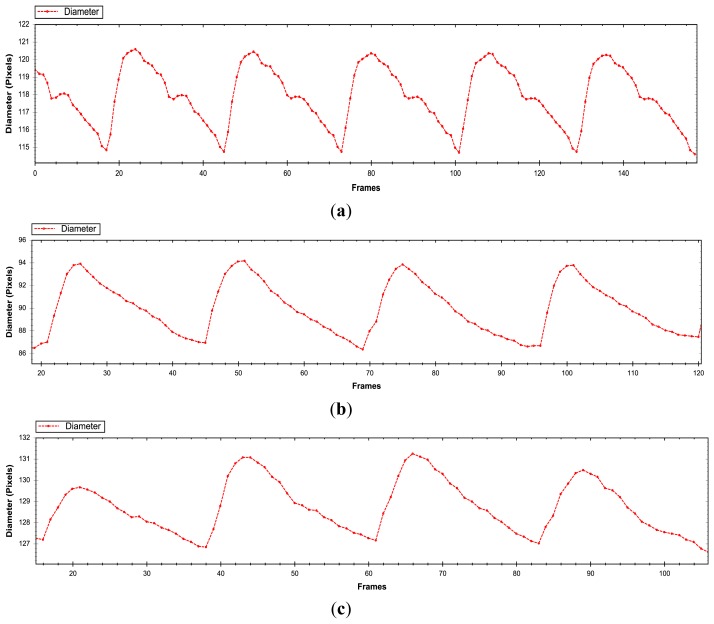
Diameter evolution of the common carotid artery (CCA) in real ultrasound data in different subjects where first row (**a**) correspond to a healthy patient and the last two rows (**b**–**c**) belong to patients with presence of atheroma plaques.

At this point, the proposed methods have been evaluated on synthetic US B-Mode imaging. To demonstrate and validate that the best approach is able to work in real sequences, it is evaluated in different subjects in the common carotid artery as shown in Figure 13. In this brief evaluation, a healthy patient (Figure 13a) was involved, whose diameter motion curves showed clearly the dicrotic peak (attributed to the elastic recoil of the aorta and aortic valve) while the other two patients (with presence of atheroma plaque) the dicrotic peak is absent (Figure 13b,c).

## 5. Conclusions

The objective of this work was the design, evaluation and comparison of methods able to characterize arterial wall motion. A set of methods has been evaluated with the objective of determining which approach better handles our problem, the estimation of the diameter of the artery over time. The motion methods were selected according to the obtained results in other works, with the goal of comparing our approach against these other methods and evaluating its accuracy. It has been demonstrated that our proposed combination of methods based on similarity transformation, non-rigid deformations, statistical filtering, and hybrid motion estimation techniques enhance existing state of the art approaches, up to 2.5 times more accurate than state of art techniques.

Synthetic US sequences with different patterns of motion were generated to allow quantitative comparative analysis of different methods and combination of techniques. Our experiments involve a total of 1620 evaluations within nine simulated sequences of 84 frames each and four error metrics. In fact, the assessment that appropriate integration of different techniques has a clear impact on the final performance represents an important contribution of this work. Another advantage that must be remarked is that the proposed methods supports large displacement vectors unlike optical flow techniques that are limited in working range and require multiscale schemes.

## Figures and Tables

**Table 1.** Wall Displacement Simulation Parameters.

**Parameter**	**Value**
*a*	15.14
*b*	−0.64
*c*	1.5
*t*_1_	0.25t
*t*_2_	0.65t
*d*	1.22
*f*	0.06, 0.12, 0.25
*T*	1

**Table 2.** Evaluated models where the used methods are indicated. Not all the combinations have been used because some of them were nonsensical.

**Methods**	**ST**	**KF**	**MS**
M1			
M2		x	
M3	x		
M4	x	x	
M5	x		x
M6	x	x	x

## Appendix: Results

**Table A1.** Longitudinal error results after being evaluated with different methods.

**Methods**	**M1**	**M2**	**M3**	**M4**	**M5**	**M6**
Lucas & Kanade	62.87	16.95	59.98	73.91	75.99	42.52
BM+ Lucas & Kanade	52.79	45.05	57.41	39.51	29.76	19.48
BM	1.10	1.15	1.68	1.68	1.27	1.28
Anisotropic Huber-L1	1.43	1.42	1.56	1.56	1.12	1.12
BM + Anisotropic Huber-L1	1.73	1.62	1.88	1.87	1.52	1.51

**Table A2.** Radial error results after being evaluated with different methods.

**Methods**	**M1**	**M2**	**M3**	**M4**	**M5**	**M6**
Lucas & Kanade	7.89	2.37	4.96	8.50	4.93	3.33
BM+ Lucas & Kanade	4.69	4.42	3.96	2.77	1.84	1.08
BM	0.52	0.88	0.63	0.64	0.64	0.64
Anisotropic Huber-L1	0.59	1.35	0.57	0.57	0.57	0.57
BM + Anisotropic Huber-L1	0.25	0.24	0.26	0.28	0.21	0.21

**Table A3.** Position Error results after being evaluated with different methods.

**Methods**	**M1**	**M2**	**M3**	**M4**	**M5**	**M6**
Lucas & Kanade	63.75	17.38	60.66	75.02	76.37	42.99
BM+ Lucas & Kanade	53.35	45.51	57.63	39.68	29.86	19.55
BM	1.30	1.49	1.80	1.81	1.49	1.50
Anisotropic Huber-L1	1.55	1.48	1.67	1.67	1.30	1.31
BM + Anisotropic Huber-L1	1.75	1.64	1.89	1.89	1.54	1.53

**Table A4.** Diameter error results after being evaluated with different methods.

**Methods**	**M1**	**M2**	**M3**	**M4**	**M5**	**M6**
Lucas & Kanade	4.36	3.08	3.79	3.70	2.52	2.89
BM+ Lucas & Kanade	9.30	5.79	2.52	2.25	1.44	1.02
BM	0.65	0.86	0.44	0.48	0.43	0.44
Anisotropic Huber-L1	0.47	0.47	0.37	0.42	0.38	0.38
BM + Anisotropic Huber-L1	0.26	0.28	0.26	0.35	0.20	0.21
